# False Negative Results in *Clostridium difficile* Testing

**DOI:** 10.1186/s12879-016-1741-6

**Published:** 2016-08-19

**Authors:** Yanal M. Murad, Justo Perez, Gustavo Ybazeta, Sarah Mavin, Sebastien Lefebvre, J. Scott Weese, Joyce Rousseau, Francisco Diaz-Mitoma, Reza Nokhbeh

**Affiliations:** 1 Current Address: Advanced Medical Research Institute of Canada, 41 Ramsey Lake Road, Sudbury, ON P3E 5J1 Canada; 2 Northern Ontario School of Medicine, 935 Ramsey Lake Road, Sudbury, ON P3E 2C6 Canada; 3 Health Sciences North, 41 Ramsey Lake Road, Sudbury, ON P3E 5J1 Canada; 4 Centre for Public Health and Zoonoses, University of Guelph, Guelph, ON N1G 2W1 Canada

**Keywords:** Clostridium difficile, Molecular testing, False negative

## Abstract

**Background:**

Accurate diagnosis of *Clostridium difficile* infection (CDI) is paramount for patient management. The wrong diagnosis places patients at risk, delays treatment, and/ or contributes to transmission of infection in the healthcare setting. Although amplification of the toxin B gene by polymerase chain reaction (PCR) is a sensitive method for detecting toxigenic *C. difficile*, false negative results still occur and could impact the diagnosis and treatment of this infection.

**Methods:**

This study investigated 48 patients that tested negative for toxigenic *C. difficile* via GeneXpert *C. difficile epi* test, while simultaneously testing positive for toxigenic *C. difficile* via stool culture. Fifty discrepant samples were collected over a 15-month period and all *C. difficile* isolates were characterized by ribotype. Patient charts were reviewed to assess whether discrepant results impacted the treatment course or clinical outcome of affected patients.

**Results:**

Fifty samples of a total of 2308 samples tested in an acute healthcare facility over a 15-month period had negative PCR and positive stool culture for toxigenic *C. difficile. C. difficile* isolated from the discrepant samples resulted in diverse ribotyping patterns suggesting they were derived from different strains. The samples belonged to patients who were distributed evenly between age groups and wards in the hospital. In the majority of cases, the false negative *C. difficile* test results did not seem to impact the clinical outcome in these patients.

**Conclusions:**

The PCR limit of detection may impact the results of molecular methods for *C. difficile* detection. Both clinical and analytical sensitivity of *C. difficile* tests should be considered when deciding which diagnostic assay to use, and clinical correlates should be examined carefully before excluding CDI as a cause of disease.

## Background

Accurate diagnosis of *Clostridium difficile* infection (CDI) is crucial for proper treatment and for preventing the spread of the pathogen in healthcare facilities. The selection of diagnostic tests to confirm CDI is controversial because of the variety of laboratory methods that are available and used across various facilities, and lack of standard recommendations for testing. The controversy is compounded by the presence of two reference methods, the cell cytotoxicity assay (CCTA), which detects *C. difficile* toxin presence directly from the stool using mammalian cell culture, and toxigenic culture (TC), in which *C. difficile* is cultured first, and the toxins in the bacterial culture supernatant are detected using the CCTA [1, 2]. Diagnostic methods for the laboratory detection of CDI can be categorized into three groups based on the detected target: 1) culture based methods, 2) methods based on detecting toxins produced by the bacteria, and 3) molecular methods based on detecting the toxin B gene (*tcdB*). Each of the three methods has its own advantages and disadvantages. Culture methods produce a bacterial culture that can be used for downstream testing (e.g. toxin gene detection, ribotyping); however, it is a time consuming method and not practical due to the long turn-around time. Molecular methods have advantages of rapid testing with short turn-around-time and high analytical sensitivity [3], but not necessarily clinical sensitivity to distinguish *C. difficile* colonization versus symptomatic CDI [4]. Many laboratories are currently using molecular methods to detect toxin B for the diagnosis of CDI [5–8], and new technologies are easy to use even as a point of care test (POCT) [9]. Molecular assays have also been shown to be superior to toxin EIAs, CCTA, and 2-step algorithms, but not to TC [10]. Immunoassay methods based on detecting toxins A and B are recognized as less sensitive for detecting toxigenic *C. difficile* (the sensitivities of these assays varied from 31.7 % to 55.2 %) [11, 12], but are still used by some laboratories as part of an algorithm for detecting toxigenic *C. difficile*. Glutamate dehydrogenase assay has been proposed as the first step in two-stage algorithms for diagnosis of CDI, but their low specificity makes them unsuitable as standalone tests [2].

The choice of an analytical method that is accurate, fast, and correlates with the clinical diagnosis of CDI can be challenging. The prevalence of asymptomatic colonization with *C. difficile* is 7 %–26 % among adult inpatients in acute care facilities [13]. This proportion of colonized carriers can reach up to 50 % in facilities where CDI is endemic [14]. Diarrhea is a sign of CDI, but many patients who are colonized with *C. difficile* may have diarrhea for reasons other than CDI. This situation may result in unnecessary treatment or initiation of strict isolation procedures in hospital [4]. The reference methods for *C. difficile* testing have different values and advantages, for example, TC identifies patients who are potentially infectious if they have diarrhea (high analytical sensitivity) while CCTA is a better test for the laboratory confirmation of CDI (having high clinical sensitivity), although further research should be conducted to confirm if CCTA sensitivity is improved with additional TC [1, 15]. Analysis of outcomes in CDI patients showed that poorer outcomes correlated with detectable free toxin in stool than positive *C. difficile* culture alone [2]. Based on the aforementioned results it would seem logical to perform highly specific rapid assays for the detection of *C. difficile* toxins in stool, however rapid assays have a sensitivity of 30–50 % [16].

In this study, we investigated a subgroup of 48 patients that tested negative by a molecular test (Cephied GeneXpert *C. difficile epi* test) while testing positive for toxigenic *C. difficile* by culture. These samples were collected between June 2012 and August 2013 and were tested retrospectively for *C. difficile* by culture. We also investigated whether the molecular test missed certain groups of *C. difficile* ribotypes or CDI patients and whether false negative *C. difficile* results had an impact on patients’ clinical outcomes.

## Methods

### Study population

All work performed in this study was approved by the Institutional Research Ethics Board (REB) of Health Sciences North (HSN) in Sudbury, Ontario, Canada. Testing for *C. difficile* by GeneXpert was part of patient care, and delegated review was given to allow for the research work. This study was conducted in a 500 bed acute healthcare facility, and comprised patients who tested negative for *C. difficile* using the GeneXpert *C. difficile epi* test, but had positive cultures for toxigenic *C. difficle*. Patients charts were analyzed during the 15-month period to account for all other occasions of *C. difficle* testing. Patient outcomes were measured based on mortality and *C. difficile* related morbidities.

### *C. difficile* testing by GeneXpert and *C. difficile* Culture

Stool samples from patients with diarrhea, and suspected of having CDI, were tested in the diagnostic laboratory for *C. difficile* using the GeneXpert *C. difficile epi* test following manufacturer’s instructions [3, 17]. *C. difficile* cultures were performed in the research laboratory. Briefly, stool samples were mixed with an equal volume of 100 % ethyl alcohol for 60 min at room temperature. One hundred microliters of the mixture were spread on *Clostridium difficile* base agar plates (CDBA) (Oxoid, Inc), supplemented with 0.1 % taurocholate (Sigma), norfloxacin 12 mg/L, moxalactam 32 mg/L, Cystein-HCl 0.5 g/L and 7 % defibrinated horse blood and incubated at 36 ± 0.5 °C for 72 to 96 h under anaerobic conditions (90 N_2_, 5 CO_2,_ and 5 H_2_). Presumptive identification as *C. difficile* was based on colony size, morphology and fluorescence properties [18]. Isolated single *C. difficile* colonies were picked and inoculated into 0.5 mL of pre-reduced Chopped Meat Glucose Broth (CMGB) (Anaerobic Biosystems, USA) and incubated for 72 to 96 h under anaerobic conditions. Bacteria and spores were collected from the broth medium by centrifugation at 13,000xg for 5 min, and the DNA was extracted by boiling the sediment in 1 % Chelex-100 resin (Sigma, Inc) suspension for 20 min. Samples were centrifuged at 13000xg for 5 min, and the supernatant was used for downstream testing.

### Multiplex PCR for identifying toxins A and B, and Ribotyping

Multiplex PCR was performed on presumptive *C. difficile* isolates to detect the Toxin A gene (*tcdA*), Toxin B gene (*tcdB*), and the triose phosphate isomerase gene (*tpi*) as described previously [19].

Ribotyping was performed using the method described by Bidet [20]. The primers used for ribotyping were 5′-GTG CGG CTG GAT CAC CTC CT-3′ (16S primer) and 5′-CCC TGC ACC CTT AAT AAC TTG ACC-3′ (23S primer). Amplification reactions were performed using Qiagen Multiplex PCR plus kit (Qiagen, CA), 50 pmoles of each primer and 100 ng of DNA. Amplification conditions for ribotyping reaction were 1 cycle of 6 min at 94 °C for denaturation; 35 cycles of 1 min at 94 °C, 1 min at 57 °C, and 1 min at 72 °C; and a final extension cycle of 7 min at 72 °C. The reactions were carried out in an Eppendorf Mastercycler® nexus.

Ribotyping amplification products were fractionated by capillary gel electrophoresis using the 2100 Bioanalyzer (Agilent Technologies). Briefly, DNA 1000 chip was prepared and used according to manufacturer’s instructions, and 1 μl of PCR product was loaded into the sample well. The chip was run using preassigned program. Data were analyzed using Gelcompar II v6.1 software (Applied Maths). Ribotyping patterns were compared to an international reference collection (Cardiff/ECDC Collection) and assigned the corresponding designation (e.g. 027). When ribotype patterns were not consistent with reference strains, internal nomenclature was assigned.

### Statistical analysis

Statistical analyses were performed using MedCalc for Windows, version 12.5 (MedCalc Software, Ostend, Belgium). Observed agreement and Kappa value were calculated using the GraphPad QuickCalcs Web site: http://graphpad.com/quickcalcs/kappa1.cfm (accessed on March 2016).

## Results

### Study population and *C. difficile* testing

During the study period, a total of 2308 samples were tested for toxigenic *C. difficle* by GeneXpert (diagnostic laboratory) and culture methods (research laboratory) over a 15-month period. The observed agreement of the results of the two tests was 95.1 % (kappa, 0.78). A total of 295 samples tested positive by GeneXpert, while 2013 samples were negative by GeneXpert.

The sensitivity, specificity, PPV and NPV for the GeneXpert method were 82, 97, 79 and 99 %, respectively when compared to the results obtained by culturing (Table 1). Toxigenic *C. difficile* was recovered by culture from 50 samples (from 48 patients) that tested negative by GeneXpert. There were 24 males and 24 females, and the average age of the patients was 64 years. Samples were collected from patients from intensive care [10], medical (16) and surgical units (8), and 14 patients from outpatient clinics. All *C. diffficle* isolates were confirmed as toxigenic *C. difficile* by multiplex PCR, and consisted of diverse group of 21 different ribotypes (Table 2).

**Table 1:** Comparison of performance of GeneXpert *C. difficile* test and *C. difficile* culture followed by toxin PCR for samples tested during the study period

		Culture	
		+	-
Xpert	+	233	62
	-	50	1963
Sensitivity	82 %		
Specificity	97 %		
PPV	79 %		
NPV	99 %		
Agreement	95 %		
Kappa	0.78

**Table 2:** Ribotypes and toxinotypes of *C. difficile* isolates recovered from 50 GeneXpert negative samples

		Toxin PCR		
Ribotype	Number of isolates	A + B+	A-B+	A(Del)B+
001	1	1		
002	3	1	1	1
020	9	6	3	
027	5	5		
046	8	6	1	1
056	2	1		1
126	3	2	1	
137	4	4		
OVC F	2	2		
Z	2	2		
Other ribotypes^a^	11	11		
Total	50	41	6	3

^a^Other ribotypes represented only once in this work include M, 017, 053, 106, S50 and 6 other unique types

### False negative PCR results

Upon examining the GeneXpert results, 40 out of the 50 samples examined in this study were negative for all 3 components of the test (ToxB, Binary toxin and the *tcdC* deletion). In 7 (14 %) of the discrepant samples, an amplification curve for toxin B target was detected by GeneXpert assay; however, the curve failed to reach the positivity threshold. In addition, 4 (8) samples were positive for the Binary toxin by GeneXpert but negative for toxin B, and another 2 (4 %) samples showed a *tcdC* deletion. Toxin gene PCR was performed on *C. difficile* isolates recovered by culture and 40 samples were A + B+, 7 samples were A-B+, and 3 samples were A-B+ but with a variant that showed a deletion in *toxA* gene (Table 2).

Ribotype 020 was the most frequent type among the 50 samples, with 9 samples making up this group. Eight samples had the ribotype 046, and 5 samples had the ribotypes 027 (Table 2). This distribution followed the general distribution of toxigenic *C. difficile* isolated from CDI patients at HSN (unpublished data).

The distribution of cases in this study reflected the relative sizes of the units, where almost one third of the cases (16 cases) originated from medical wards. Fourteen cases were outpatients, and the rest of cases were distributed between surgery patients and intensive care units patients.

### Clinical features

When we examined the charts for the 48 patients from this study, 16 patients were tested for *C. difficile* only once over the study period, while another 16 patients were tested more than once, but were consistently negative by GeneXpert *C. difficile epi* test. For these 32 patients, there was no chance of identifying them as *C. difficile* positive by GeneXpert test.

On the other hand, 16 patients tested positive by GeneXpert *C. difficile epi* test on other occasions. Of these, 8 patients tested positive in other occasions (4 tested positive before and 4 after the negative test). The other 8 patients tested both positive and negative by GeneXpert *C. difficile epi* test on multiple occasions (with 5 patients having the first test on record as positive and 3 as negative) (Table 3). For the patients that were tested multiple times with mixed results, the patients’ records indicated clearly that these patients had a history of CDI.

**Table 3:** The breakdown of results of other GeneXpert tests (if patients have been tested more than once)

Number of patients	Test description	First test negative	First test positive
16	Patients tested only once	16	0
16	Patients Tested Negative on other occasions	16	0
8	Patients Tested Positive on other occasions	4	4
8	Patients Tested both Positive and Negative on other occasions	3	5

GeneXpert negative/ Culture positive (50 samples from 48 patients or 2.2 % of total testing volume)

For the 16 patients that were tested only on one occasion for *C. difficle*, there was no chance of diagnosing these patients with CDI, but their chart review revealed that only one patient presented with a GI related illness, and who had a history of ulcerative colitis. Another patient died from an unrelated infection.

Only five of the 48 patients in this study were admitted with gastrointestinal diseases or disorders. One of the five patients was diagnosed with CDI, and this patient had a positive GeneXpert *C. difficile* test on another occasion during the admission. The other 4 patients had a GI malignancy (2 patients), Crohn’s disease (1 patient) or ulcer of the oesophagus (1 patient).

There were 16 patients who tested positive for *C. difficile* by GeneXpert on stool tests taken at different times. Eight of these 16 patients had another positive sample tested during the same episode of disease. Five of these patients had at least one previous positive test for *C. difficile* before this episode. The remaining patients (3) were tested positive at a later time.

When we examined the mortality rate among these 48 patients, 10 of them died in the hospital, with 4 deaths due to respiratory illnesses, 2 due to CNS disease, 2 infections, 1 cancer, and 1 cardiac disease. The clinical outcome of these patients did not seem to be associated with CDI.

## Discussion

Choice of optimal tests is based on various factors, including sensitivity, specificity, cost and turnaround time. Real time PCR (GeneXpert) has been used at this facility as the primary CDI test, and while agreement between GeneXpert and culture was high, toxigenic *C. difficile* was recovered from 50 samples that tested negative by GeneXpert assay (2.2 % of the total samples that were tested by GeneXpert *C. difficile epi* test over the study period).

In this study, we used a two-step test (culture followed by PCR) to isolate and identify *C. difficile* from stool samples. This method is labor intensive and requires days to produce results, thus it is not suitable for use in clinical laboratory. However, toxin PCR and ribotyping data obtained are very useful to track and study CDI cases. Laboratory tests used to diagnose CDI were discussed in the introduction, and many molecular methods use the *tcdB* as a target for detecting toxigenic *C. difficile* directly from stool sample. One of the newer methods for analysing *C. difficile* is by using MALDI-TOF, which is able to identify and differentiate between different riboytpes [21, 22], however to use this method, *C. difficile* must be recovered by culture, which would not be feasible for a diagnostic laboratory.

The false negative results obtained in the subset of GeneXpert *C. difficile* samples might have been due to low levels of *C. difficile* that have fallen below the level of detection by PCR. This observation (toxigenic *C. difficile* present at or below the limit of detection of the Xpert® *C.difficile* assay) has been reported previously in a study evaluating the use of GeneXpert for detection of environmental contamination with *C. difficile*, where only one-third of environmental specimens positive by toxigenic *C. difficile* culture were detectable as positive by the GeneXpert assay [23]. Another study examined the indeterminate results for *tcdB* GeneXpert *C. difficile* PCR assay, which are defined as the detection of a typical PCR amplification curve for *tcdB* gene with an Ept >10 that was interpreted as negative by the GeneXpert® assay [24]. This study found that at least one third of these results are associated with positive *C. difficile* culture [24].

A recent study has shown an association between *C. difficile* fecal load and the results of routinely used diagnostic tests [25]. The authors concluded that the association between bacterial load and the results of ToxAB and CCTA is likely indirect, as these latter assays detect the presence of toxins rather than the bacteria *per se* [25]. Unfortunately, this study examined only PCR positive samples, which would exclude any false negative samples that might have been missed by PCR [25]. Other studies that compared the GeneXpert *C. difficile epi* test to culture methods has shown a sensitivity, specificity, PPV and NPV of 98.8, 90.8, 56.6, and 99.8 % respectively, while the values were 93.5, 94.0, 73.0, and 98.8 % when enriched TC was used [5]. Our data shows a higher number of false negative samples compared to this study. As mentioned before, a false negative *C. difficile* test might be related to the limit of detection of the assay. An additional explanation of the results was provided by another study that was performed to examine patients who tested positive for *C. difficile* after repeated PCR. This study found that patients who test positive for *C. difficile* by PCR within 7 days of a negative test are more likely to have a history of CDI than are patients who test negative with repeat PCR [26]. This scenario has been seen in at least 5 cases in our study. Due to the high sensitivity and nature of the PCR assays in general, which can detect DNA material even in the absence of viable bacterial cells or spores, it is more likely to encounter false positive PCR results for C. difficile when compared to culture method.

Due to the high sensitivity of molecular testing for *C. difficile*, repeated testing or testing as a proof of cure is usually not recommended. During the time of this study, repeated samples were not rejected by the diagnostic lab, and these samples were tested by both GeneXpert and culture methods. This provided valuable information on how the test performed on these repeated samples. Close to 32 % of samples described in this study came from patients that were tested only once during the study period for *C. difficile*. The rest of the samples were tested multiple times, sometimes with mixed results (summarized in Table 3). In several instances, we have seen patients that have produced both positive to negative results several times. *C. difficile* isolated from these patients have consistently produced the same ribotype. There is a possibility that these patients were re-infected with *C. difficile* of the same ribotype, but given the genetic diversity of the isolates we encountered at HSN, this possibility was not strong. Rather, the stronger possibility indicates that these patients consistently carried the same *C. difficile* strain, even when *C. difficile* could not be isolated from stool. Treatments given to CDI patients have been also shown to affect the *C. difficile* test results [27].

The other significant aspect of this study is that all patients tested with false negative results had diarrhea or liquid stools. Misdiagnosing symptomatic patients who have diarrhea may result in missing the chance to initiate treatment earlier and isolating these patients, thus increasing the chances of transmitting the bacterium to other patients.

## Conclusion

In conclusion, although molecular methods are considered among the most sensitive methods available for clinical diagnosis of CDI, it seems inevitable that some cases which are positive for *C. difficile* might be missed when using this method, or any other method for that matter. Even though in most of the cases that were reviewed in this work, the presence of *C. difficile* did not seem to contribute to the clinical picture or the illness of the affected patients (either the *C. difficile* was not producing significant symptoms, or the clinical picture of these patients didn’t seem affected by the presence of *C. difficile*), it would be reasonable to suggest that clinicians always keep in mind that any diagnostic method, no matter how sensitive, can miss sometimes. Being aware of the patient’s symptoms, and considering a repeat test for *C. difficile*, if CDI is still suspected as a cause of illness might help in identifying patients with CDI.

## Abbreviations

CCTA, cell cytotoxicity assay; CDBA, *Clostridium difficile* base agar; CDI, *Clostridium difficile* infection; EIA, Enzyme Immunoassay; Ept, end-point; NPV, Negative Predictive value; PPV, Positive Predictive Value; TC, toxigenic culture; *tcdB*, Toxin B gene
